# PC12 Cell Line: Cell Types, Coating of Culture Vessels, Differentiation and Other Culture Conditions

**DOI:** 10.3390/cells9040958

**Published:** 2020-04-14

**Authors:** Benita Wiatrak, Adriana Kubis-Kubiak, Agnieszka Piwowar, Ewa Barg

**Affiliations:** 1 Department of Basic Medical Sciences, Wroclaw Medical University, 50-556 Wroclaw, Poland; ewa.barg@umed.wroc.pl; 2 Department of Toxicology, Wroclaw Medical University, 50-556 Wroclaw, Poland; adriana.kubis-kubiak@umed.wroc.pl (A.K.-K.); agnieszka.piwowar@umed.wroc.pl (A.P.)

**Keywords:** PC12, NGF, differentiation, coating, collagen, polylysine, neurons

## Abstract

The PC12 cell line is one of the most commonly used in neuroscience research, including studies on neurotoxicity, neuroprotection, neurosecretion, neuroinflammation, and synaptogenesis. Two types of this line are available in the ATCC collection: traditional PC12 cells grown in suspension and well-attached adherent phenotype. PC12 cells grown in suspension tend to aggregate and adhere poorly to non-coated surfaces. Therefore, it is necessary to modify the surface of culture vessels. This paper aims to characterise the use of two distinct variants of PC12 cells as well as describe their differentiation and neuronal outgrowth with diverse NGF concentrations (rat or human origin) on various surfaces. In our study, we evaluated cell morphology, neurite length, density and outgrowth (measured spectrofluorimetrically), and expression of neuronal biomarkers (doublecortin and NeuN). We found that the collagen coating was the most versatile method of surface modification for both cell lines. For adherent cells, the coating was definitely less important, and the poly-d-lysine surface was as good as collagen. We also demonstrated that the concentration of NGF is of great importance for the degree of differentiation of cells. For suspension cells, we achieved the best neuronal characteristics (length and density of neurites) after 14 days of incubation with 100 ng/mL NGF (change every 48 h), while for adherent cells after 3–5 days, after which they began to proliferate. In the PC12 cell line, doublecortin (DCX) expression in the cytoplasm and NeuN in the cell nucleus were found. In turn, in the PC12 Adh line, DCX was not expressed, and NeuN expression was located in the entire cell (both in the nucleus and cytoplasm). Only the traditional PC12 line grown in suspension after differentiation with NGF should be used for neurobiological studies, especially until the role of the NeuN protein, whose expression has also been noted in the cytoplasm of adherent cells, is well understood.

## 1. Introduction

Recent neurotoxicity tests on nervous system diseases rely mostly on animal models. These experiments are associated with ethical questions as well as are expensive, laborious, and often produce vague results [1]. In vitro methods, on the other hand, are relatively fast and less demanding ways to test chemicals for their neurotoxic properties [1]. In vitro techniques to study neurodegeneration are based principally on chemical-induced effects in cell lines derived from rodent or human, and allow one to uncover basic, specific impacts on individual cells or relevant molecular pathways [2].

Nevertheless, proper design of the experiment, meaning the suitability of a cell model that will be optimal for responding to a particular hypothesis, relies principally on the right choice of the cell line. The use of an inappropriate model may lead to an incorrect assessment of neurotoxic properties. Moreover, the environment in which the cell culture is maintained can influence their intrinsic characteristic, therefore, changing the final results of performed experiments.

Rat pheochromocytoma cells PC12 offer an extensively used model in neurobiology as they exhibit some features of mature dopaminergic neurons [3]. Although these cell lines originate from a pheochromocytoma of the rat adrenal medulla, they have been extensively characterised for neurosecretion (catecholamines, dopamine, and norepinephrine) and the presence of ion channels and neurotransmitter receptors [4]. The popularity of PC12 cells is mainly due to their extreme versatility for pharmacological manipulation, ease of culture and a large amount of background knowledge on their proliferation and differentiation. PC12 cells grown under normal conditions are characterised by morphology, physiology, and biochemistry of the adrenal cells. When cultured in the presence of nerve growth factor (NGF), they differentiate into sympathetic ganglion neurons morphologically and functionally [5,6]. PC12 cells synthesise and store dopamine and are a suitable model for the study of catecholaminergic neurotoxicity in vitro.

Widespread use of PC12 cells is to study the neurotoxic activity of various substances, for example, by assessment of the effect on cell survival, neurite outgrowth, DNA damage or protein expression levels. However, this cell line is also widely used as a model for neurodegenerative diseases. Alzheimer’s disease can be modelled by the exogenous administration of β-amyloid peptide [7], and Parkinson’s disease by inducing damages using 1-methyl-4-phenylpyridinium or 6-hydroxydopamine [8]. Research on amyotrophic lateral sclerosis can be conducted using PC12 with mutated SOD1 gene (G93A) [9], and ischemic stroke can be simulated by oxygen and glucose deprivation [10]. By using these models with an artificially induced pathological condition, PC12 cells are very often used to study the protective or regenerative impact of various substances of natural or synthetic origin on neuronal cells.

NGF-differentiated PC12 cells express the synapsin I protein, which is a marker of synaptic communication. Furthermore, the level of this protein expression has been proven to depend on the level of cell differentiation [11]. Synapsin I is involved in the release of neurotransmitters in synapses, but it has also been shown to play a significant role in synaptogenesis and neuronal plasticity [12]. An interesting work demonstrating the potential of PC12 cells in the analysis of neurotoxicity and synaptogenesis was performed by Bernardes et al. [13]. Besides other markers, authors have studied the levels of the expression of synapsin I in cells treated with neurotoxin acrolein possibly connected with the development of Alzheimer’s disease. Another example of such work is the paper about neurotrophic and neuroprotective effects of caffeic acid phenethyl ester against cisplatin-induced neurotoxicity [13].

There are also many works showing that PC12 cells can be used in more complex neurobiological models, including using co-cultures with other types of cells. Due to the presence of TLR4 (Toll-like receptor 4) receptors, PC12 cells are used, among others for the study on the neuroinflammation, especially through co-cultures with microglia or astrocytes (where the response is enhanced by proinflammatory cytokines IL-1 and IL-6) [14,15].

Differentiated PC12 cells can form functional synapses with each other as well as with other neuronal cells [16,17]. This enables the PC12 line to be used in mixed cultures for research into synapse formation. Such studies use co-cultures of primary neuronal cells and other cells transfected with cDNA encoding the appropriate synaptic adhesion molecule to be tested. The HEK 293 or the Chinese hamster ovary (CHO) cells are usually used for this purpose, but the PC12 cell line may have some benefits due to the endogenous expression of many neuronal proteins [18,19].

**Figure 1:**
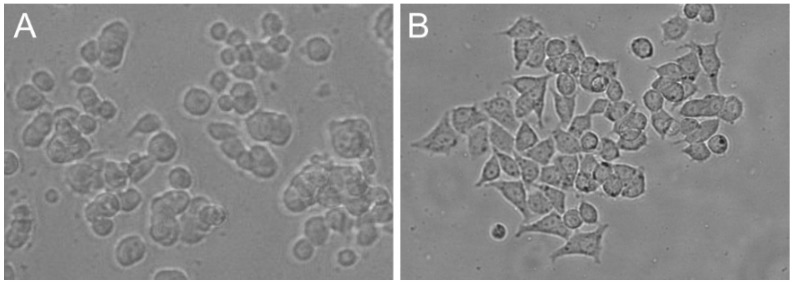
PC12 (**A**) and PC12 Adh (**B**) cells in culture bottles.

There are many kinds of PC12 cells reported in scientific papers. In American Type Culture Collection (ATCC) there are distinguished two variants of this cell line—traditional PC12 cells (PC12, ATCC CRL-1721) that grow in suspension and well-attached adherent phenotype (PC12 Adh, ATCC CRL-1721.1) that exhibit an increased growth rate [20,21]. The suspension PC12 cells grow as small, irregularly shaped, floating cell clusters or as a few scattered lightly attached cells. They tend to aggregate and adhere poorly to non-coated surfaces. On the other hand, PC12 Adh cells have a good ability to adhere to plastic surfaces. An adherent variant of the cell line was isolated by repeated culturing on Corning’s CellBIND flasks [20]. The comparison of differences between those two lines is presented in Table 1 and Figure 1. In some studies, the researchers found that not every PC12 cell is appropriate for every experimental model [22,23]. ATCC has performed experiments on neurite outgrowth in response to neuronal growth factor (NGF) treatment (0.2 µg/mL) on collagen-coated plates. After three days of incubation with NGF, the development of new neurites was observed.

Despite the challenges associated with selecting the most appropriate cell line, another obstacle can be related to the influence of the cell’s surrounding environment on the course of the experiment [26]. Environmental factors affecting the growth of animal cells include, among others, optimal medium selection, the temperature of incubation as well as suitable plastic multi-well plates used in experiments, finishing with surface modification of cell culture plates [27,28,29,30]. The appropriate coating helps to overcome the problem of poor cell adhesion to culture vessels [31]. Various surface coating protocols have been developed to skip this technical limitation in designing experiments [32]. For many cell lines, and especially for post-mitotic neurons, coated culture plates are a prerequisite for seeding. Commonly used coating reagents are, among others, poly-l-lysine (PLL), poly-d-lysine (PDL), fibronectin, laminin, and collagen [33,34].

This paper aims to characterise the use of two distinct variants of PC12 cells as well as describe their differentiation and neuronal outgrowth with diverse NGF concentrations on various surfaces. There are currently relatively few reports available on the use of the PC12 Adh line [35,36]. To our knowledge, this is the first paper that describes differences in the methodological approach to non-adherent and adherent variants of the PC12 cell line. The purpose of this article is also to advise how to choose the proper type of PC12 cells and experimental conditions in such a way that they will optimally respond to the experimental hypothesis. Cell morphology, neurite lengths, density and outgrowth, and expression of neuronal biomarkers were evaluated. As an additional element of the study, the effect of various concentrations of NGF (50 or 100 ng/mL) and its origin (rat or human) on the differentiation of PC12 and PC12 Adh cells was compared.

## 2. Materials and Methods

### 2.1. Cell Lines

The PC12 (CRL-1721) and PC12 Adh (CRL-1721.1) cell lines were obtained from ATCC. Both cell lines were cultured at 37 °C, 5% CO_2_ and passaged twice a week, and the experiments were performed on cells between passage 15 and 25.

After removing the supernatant from PC12 Adh cells, they were washed with PBS, then treated with TrypLE Express solution (Gibco, Thermo Fisher Scientific, Waltham, MA, USA, cat. no. 12604-021) and incubated for 5 min at 37 °C. Next, the cells were transferred to a centrifuge tube, complete medium was added (to inactivate the TrypLE Express), and cells were centrifuged for 5 min at 1000 g. The supernatant was removed, and cells were resuspended in fresh medium.

PC12 cells grown in suspension were transferred to a tube and centrifuged under the same conditions as PC12 Adh. After the supernatant decantation and addition of fresh medium, cell clumps were broken using 20 mL syringe with a needle. If the cells were taken for assays, 22 and 25-gauge needles were used (each needle size was used three times from the largest to the smallest diameter). However, if the cells were only passaged, only an 18-gauge needle was used twice.

For immunocytochemical staining and a spectrofluorimetric neurite outgrowth assay, cells were seeded at a density of 1 × 10^5^ cells/well in 96-well culture plates, while 1000 cells/well cell density was used for the differentiation experiments. After plating, the cells were left for 24 h for adhesion to the surface of the culture vessel.

### 2.2. Culture Medium

Two different culture media were used in the study: complete medium in which cells were cultured and the second medium to differentiate cells (differentiation medium). The complete medium consisted of RPMI-1640 (Lonza, Basel, Switzerland, cat. no. BE12-702F) supplemented with 10% donor horse serum (DHS; Biological Industries, Beit-Haemek, Israel, cat. no. 04-004-1A), 5% fetal bovine serum (FBS; Biological Industries, cat. no. 04-001-1A), 2 mM L-glutamine (Lonza, cat. no. 17-605E), 25 µg/mL gentamicin (Lonza, cat. no. 17-518L) and 2.5 µg/mL amphotericin B (Gibco, cat. no. 15290026). The differentiation medium was also based on RPMI-1640 with L-glutamine, gentamicin, and amphotericin B, but only with 1% DHS and without FBS, and with the addition of nerve growth factor (NGF). There were used two forms of NGF: rat NGF (Sigma-Aldrich, Saint Louis, MO, USA, cat. no. N2513) and recombinant β-NGF of human origin (Sigma-Aldrich, cat. no. SRP3015) both at concentrations of 100 ng/mL. The culture medium was changed every 48 h.

### 2.3. Cell Culture Plate Coating

The surface of the wells of microplates has been modified to allow adhesion of PC12 cells. For this purpose, poly-l-lysine (PLL, Sigma Aldrich, cat. no. P5899), poly-d-lysine (PDL, Sigma Aldrich, cat. no. P6407) and type I collagen (Sigma Aldrich, cat. no. C9791) solutions were used. PDL is a synthetic substance, and PLL and type I collagen occur naturally [34,37]. These are the most popular methods of surface modification based on data from scientific publications.

Both PLL and PDL were dissolved in distilled water to achieve 10 mM stock solution, whereas type I collagen was dissolved in 0.1 M acetic acid to a concentration of 0.1% (*w*/*v*). The prepared stock solutions were stored at −20 °C for up to 6 months. Working solutions were prepared briefly before use as follows. The PLL and PDL were diluted to a final concentration of 0.1 mM in distilled water and type I collagen solution was prepared by diluting the stock solution in distilled water 10 times. Each final solution was added to the well in the volume needed to cover the surface. PLL and PDL were left for 30 min at RT, and plates with collagen solution were put at 4 °C overnight. After incubation, the solutions were removed, and all plates were washed three times with 200 µL of PBS for 5 min. PLL or PDL coated plates were stored at RT, while collagen-coated plates were placed at 4 °C and stored for up to one month.

### 2.4. Evaluation of Cell Culture Plate Coatings

Cell culture plate coatings made of type I collagen, PLL, and PDL were evaluated based on PC12 cells (grown in suspension) morphology. Differences in the morphology on each coated surface were assessed 24 h after cell seeding. For this purpose, the cells were classified into two groups, according to the degree of cytoplasm reorganisation. The first group included cells whose bodies had a circular shape. The second group consisted of flattened cells of polygonal shape in which cytoplasm reorganisation took place. Analyses were performed for 5 independent experiments, each of which consisted of 3 replicates. For each replicate, the first 100 cells observed were classified into one of the groups.

### 2.5. Length and Density of Neurites

Average length and density of neurites were evaluated based on microscopic images 24 h after seeding (day 0) and then on days 2, 3, 5, 7, 14, and 21 of incubation with 100 ng/mL rat NGF. In these assays, cells were seeded with reduced density (1000 cells/well). Cell differentiation using NGF results in a dense network of neurites and fewer cells have facilitated the analysis of neurite belonging to individual cells.

Density and lengths were determined for 50 cells in 3 different wells in 5 independent replicates using the ImageJ software. We measured neurites whose length was at least twice as long as the body length of the cell. Neurite density is understood as the average number of neurites per cell.

### 2.6. Neurite Outgrowth (Spectrofluorimetric Evaluation)

Neurite outgrowth is one of the most important parameters allowing to assess the morphological phenotype of neuronal cells, correlated with their proper functioning and condition. “Neurite Outgrowth Staining Kit” (Invitrogen, Thermo Fisher Scientific, Carlsbad, CA, USA, cat. no. A15001) was used for spectrofluorimetric assessment of neurite outgrowth. This assay was performed on day 3 (this incubation time was chosen based on other assays) to compare the effect of surface modification and NGF administration—neurite outgrowth was measured on collagen and non-coated surfaces, with or without NGF addition.

After removal of the supernatant, the cells were washed with PBS, and the staining solution was added to each well. Cells were incubated for 20 min at 37 °C. After incubation, cell cultures were washed with PBS, a background suppression dye was administered, and a spectrofluorimetric measurement was performed at 485/535 nm (excitation/emission wavelength) using Varioskan LUX microplate reader (Thermo Scientific). Cultures were also analysed under an EVOS FL microscope (Thermo Fisher Scientific) with a fluorescence filter.

### 2.7. Expression of Neuronal Biomarkers

The expression of doublecortin (DCX) and NeuN proteins, which are neuronal markers, was evaluated on day 3 of incubation (selected based on other assays) in collagen-coated culture plates. The purpose of this test was to check whether these biomarkers are expressed for both lines.

The assay was performed by immunostaining methods according to standard procedures. Cells were fixed with 100% cold methanol for 5 min and then washed with PBST (0.1% TWEEN 20 in PBS) three times (5 min). The cell membrane was permeabilised using a solution of 0.1% Triton X-100 in PBS for 10 min at RT. After blocking of non-specific antibody binding with a solution containing 1% bovine serum albumin (BSA) and 10% normal goat serum (NGS) in PBST for 30 min, cells were incubated with anti-NeuN antibody conjugated with Alexa Fluor 488 (Abcam, Cambridge, UK, cat. no. ab190195) and doublecortin (DCX) antibody conjugated with phycoerythrin (Novus Biologicals, Littleton, CO, USA, cat. no. NBP1-92684PE) for 1 h at RT. The concentration of NeuN antibody was 1:100 and DCX 1:500 in PBST with 1% BSA. After washing the culture, it was preserved in mounting medium. The results were analysed with fluorescence microscopy.

### 2.8. Statistical Analysis

At least three independent experiments were performed for all assays. Due to a large number of replicates, parametric statistical tests were used. Statistical analysis of data was performed with Statistica 13 software using ANOVA followed by post hoc Tukey’s test. Results where *p* < 0.05 was considered to be statistically significant. All results in the graphs are presented as mean ± SEM.

## 3. Results

### 3.1. Evaluation of Cell Culture Plate Coatings

**Figure 2:**
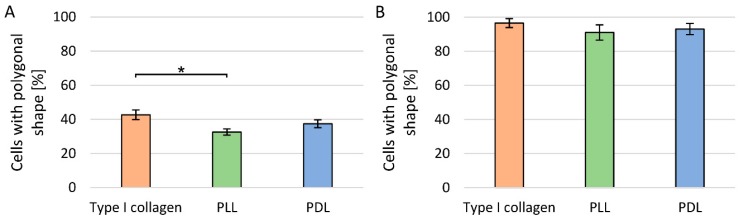
Evaluation of cell morphology for different types of surface coatings of culture plates: (**A**) PC12 cell line; (**B**) PC12 Adh cell line; * *p* < 0.05—significant difference between coating types.

The largest number of polygonal PC12 cells were detected on the surface modified with type I collagen, and the smallest for the surface modified with PLL. The difference between the number of PC12 cells with a polygonal shape on the collagen and PLL surfaces was statistically significant. In contrast, PC12 Adh cells, regardless of the type of surface modification or its absence, have a polygonal shape after 24 h of adhesion (Figure 2).

### 3.2. Length of Neurites

**Figure 3:**
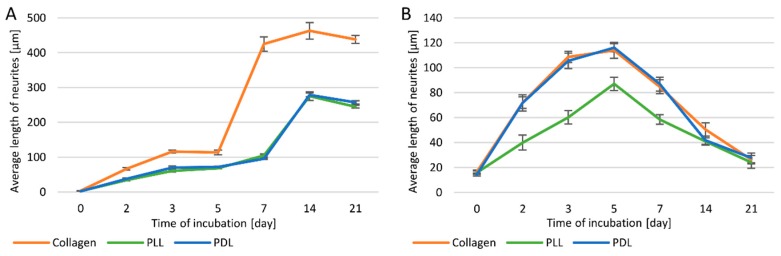
The average length of neurites in PC12 (**A**) and PC12 Adh (**B**) cells.

For both PC12 and PC12 Adh cells, the highest average length of neurites was also observed for the surface modified with type I collagen (Figure 3A,B).

Comparing the average neurite length between two cell lines (Figure 3A,B), it was found that only on days 3 and 5 on collagen and PDL coatings, and on day 5 on PLL coating longer neurites were observed for PC12 Adh cells (by about 43 μm) compared to the traditional PC12 line. In contrast, in the following days, neurites were definitely longer in PC12 cells. In general, suspension cells have the ability to form longer neurites compared to PC12 Adh cells.

Between days 5 and 7 of the study, there was a substantial increase (by 268 µm) in neurite length in traditional PC12 cells in cultures on the collagen surface, while the increase on other surfaces was much smaller (23–36 µm). On the other hand, a strong increase in the length of neurites was observed for cultures on PLL and PDL surfaces between 7 and 14 days (by 170–183 µm), while the difference on the collagen surface was not prominent (by 33 µm). However, the most elongated neurites on each surface were observed for the PC12 cells on day 14 of the analysis (collagen—114 µm, PLL—87 µm, PDL—116 µm). In all cases, on day 21 of the study, neurites were already shorter, but the decrease in length was not significant. On collagen, neurites were longer after 7 days of incubation, even compared to the culture on other surfaces on day 14.

For adherent cells, from day 2 to 7 of incubation, a significantly higher average neurite length was observed in cultures on collagen or PDL modified surfaces compared to PLL. The effects of using collagen and PDL were almost identical. For all coatings, the longest neurites were on the 5th day of incubation, and in the following days a relatively rapid decrease in length was noted, and cell division and increased proliferation were again observed.

**Figure 4:**
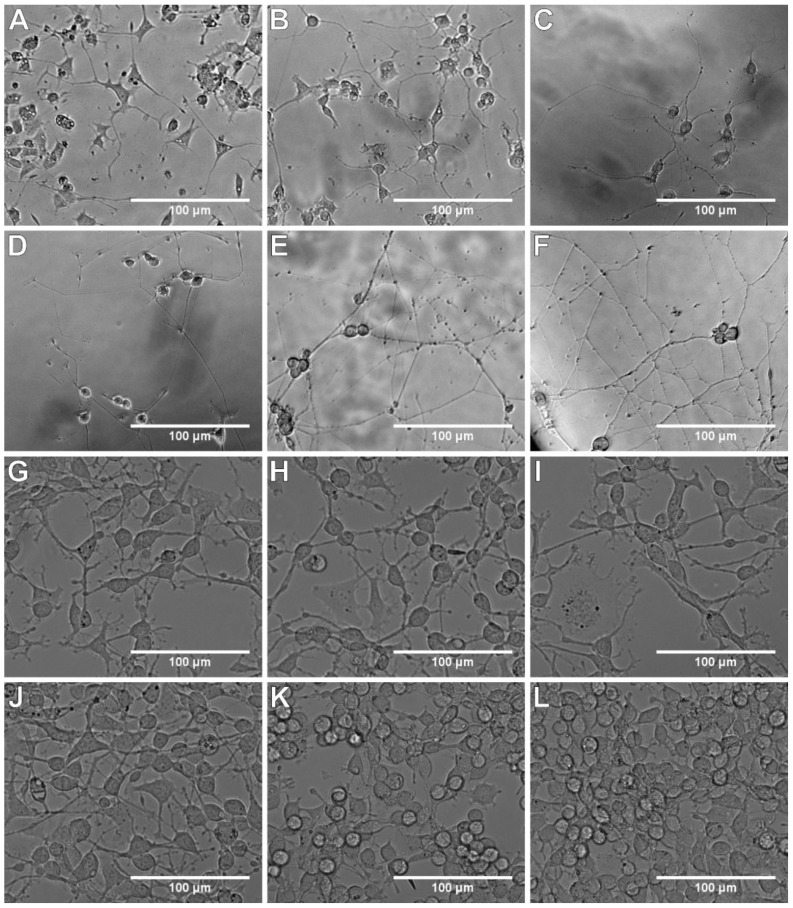
Microphotographs of cell cultures on subsequent days of neurite assessment: (**A**–**F**) PC12 cell line; (**G**–**L**) PC12 Adh cell line; (**A**,**G**) 2 days of incubation with nerve growth factor (NGF); (**B**,**H**) 3 days; (**C**,**I**) 5 days; (**D**,**J**) 7 days; (**E**,**K**) 14 days; (**F**,**L**) 21 days.

Sample microphotographs of cell cultures of both PC12 lines on subsequent days of neurite assessment are presented in Figure 4.

### 3.3. Neurite Density

**Figure 5:**
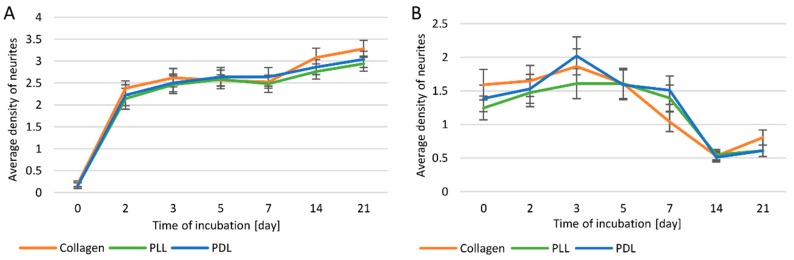
Neurite density in cells of PC12 (**A**) and PC12 Adh (**B**) lines.

Neurite density was much higher in suspension cells compared to adherent cell line. In addition, a continuous increase in neurite density was observed for the traditional PC12 line during the study (Figure 5A). In the case of the PC12 Adh line, the dependence of neurite density on incubation time was similar as for neurite length, i.e., the maximum level was reached on day 3, and later with each measurement, there was a decrease in density (Figure 5B). Adherent cells proliferated on days 14 and 21, and almost no neurites were observed despite continuous treatment with 100 ng/mL NGF. No statistically significant differences in neurite density were observed depending on the surface modification method for both PC12 and PC12 Adh cells.

### 3.4. Neurite Outgrowth (Spectrofluorimetric Evaluation)

The rest of the study was carried out for only one selected incubation time with NGF and one method of surface modification. An incubation time of 3 days was chosen due to the fact that in suspension cells the length and density of neurites on days 3 and 5 were similar, and the degree of differentiation of the PC12 Adh cells was also high on these days.

Neurite outgrowth in both PC12 cell lines was measured spectrofluorometrically for cultures on collagen and non-coated surfaces, with or without NGF addition. Collagen coating was used as this method was most beneficial for both cell lines.

**Figure 6:**
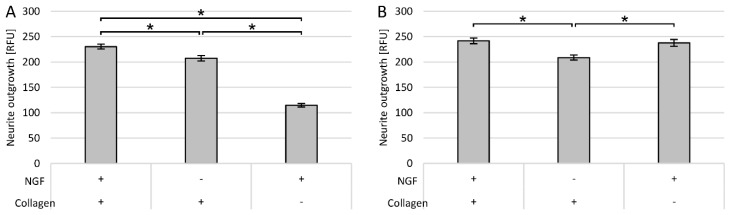
Neurite outgrowth on collagen and non-coated surfaces, with or without NGF addition, measured spectrofluorimetrically (RFU—relative fluorescence unit): (**A**) PC12 cells; (**B**) PC12 Adh cells; * *p* < 0.05—significant difference in neurite outgrowth.

As expected, the highest fluorescence was observed for cultures with NGF on the collagen surface (Figure 6A,B). In the case of traditional PC12 cells, the measured neurite outgrowth was statistically significantly larger compared to culture on collagen without NGF and with NGF but on the non-modified surface (Figure 6A). For PC12 Adh cells, the type of surface was not significant, and the same level of outgrowth was also noted on the non-modified surface (Figure 6B). For both lines, the lack of NGF administration resulted in a statistically significantly lower measurement result, but interestingly, in the case of suspension cells, the surface used was much more critical than NGF treatment application.

### 3.5. Expression of Neuronal Biomarkers

**Figure 7:**
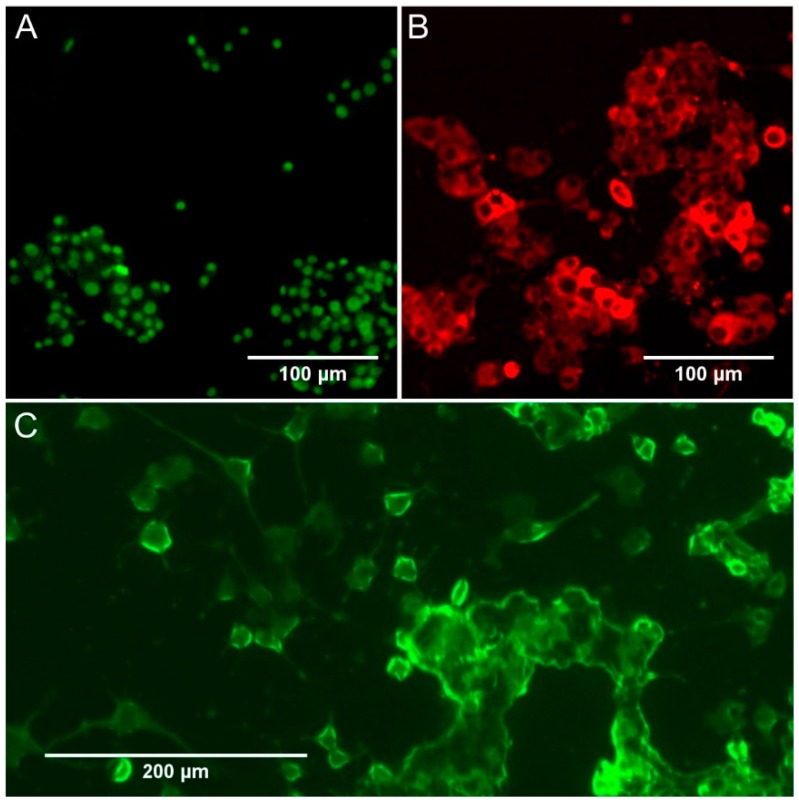
Micrographs showing the expression of neuronal biomarkers: (**A**) NeuN expression in PC12 cells; (**B**) doublecortin (DCX) expression in PC12 cells; (**C**) NeuN) expression in PC12 Adh cells.

Expression of both doublecortin (DCX) and NeuN proteins was observed in the traditional PC12 cell line (Figure 7A,B). In contrast, only NeuN expression was noted in the adherent line (Figure 7C). The DCX protein is characteristic for developing neurons, while the NeuN protein has been considered for a long time as a biomarker of adult cells of this type.

A substantial difference was observed in the NeuN expression between PC12 and PC12 Adh cells. In traditional PC12 cells, expression was localised to cell nuclei. In contrast, in adherent cells, expression was observed in the entire cell—both in the nucleus and the cytoplasm. Expression of NeuN outside the cell nucleus cannot be considered a neuronal marker because it has been reported for cells derived from many different organs (other than the brain), as well as many cell lines known to be non-neuronal cells [38]. The quantitative comparison was not performed due to the expression of the evaluated biomarkers in various cellular structures (the quantitative assessment would be unreliable).

### 3.6. Effect of NGF Origin and Concentration

As the last additional step of the study, a comparison of the activity of two different NGF concentrations of different origin was made. By assessing the average length and density of neurites, the effect of NGF of human and rat origin was examined at concentrations of 50 or 100 ng/mL.

**Figure 8:**
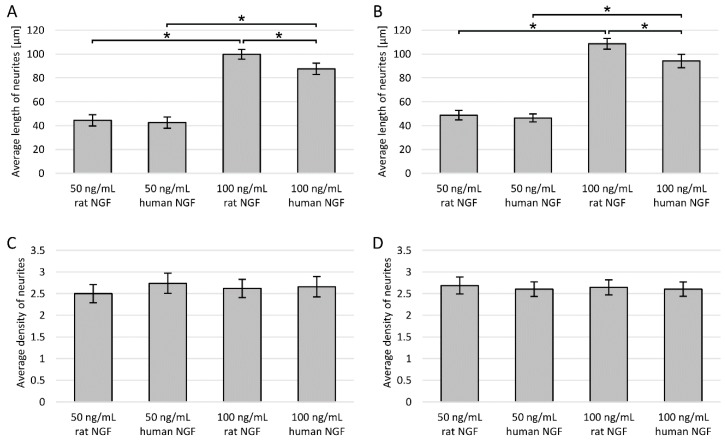
Neural features after NGF administration of various origin and in different concentrations: (**A**) length of neurites in PC12 cells; (**B**) length of neurites in PC12 Adh cells; (**C**) neurite density in PC12 cells; (**D**) neurite density in PC12 Adh cells; * *p* < 0.05—significant difference in length of neurites.

It has been confirmed that the NGF administration increases the average length and density of neurites (Figure 8). For both cell lines, the determined neurite density was almost identical, regardless of the concentration and type of NGF (Figure 8C,D). However, in the case of neurite length assessment, the difference depending on the concentration was very pronounced and statistically significant—neurites were about two times longer at the concentration of 100 ng/mL for both cell lines (Figure 8A,B). In addition, there was a relatively small but statistically significant difference between rat and human NGF at 100 ng/mL, and cells treated with rat NGF had longer neurites.

## 4. Discussion

The PC12 cell line derived from rat pheochromocytoma is an immortalised cell line similar to the primary culture of fetal neurons. They are relatively easy to passage and culture and have some features of neurons, which makes them useful in the study of nerve physiology and pharmacology. There are two collections of the PC12 line: Chinese (weak, medium and highly differentiated) and ATCC (American Type Culture Collection) including PC12 cells grown in suspension and adherent PC12 Adh cells [3]. PC12 cells are a type of catecholamine cells that synthesise, store and release norepinephrine and dopamine. There are significant morphological and growth differences between the PC12 and PC12 Adh cell lines. In the scientific community, it is believed that the PC12 Adh line is only appropriate for conducting experiments with ROCK inhibitors. In contrast, the suspension cells under the influence of nerve growth factor (NGF) resemble the neurons of the cortex—its use is recommended in studies of the physiology and pathologies of the nervous system [3].

Suspension PC12 cells have a round shape with a diameter of 6–14 μm and form aggregates. These cells require to adhere to the surface before differentiation. However, they do not adhere to the plastic surface, so it is necessary to modify the surface of the culture vessels [39]. The PC12 cells grow in suspension up to about 30 passage. Then morphological changes of cells are observed, and some begin to adhere to plastic surfaces. Kinarivala et al. have shown that a change in passage during the study can cause errors in the results [40]. It has been shown that PC12 cells without differentiation with a high passage number are less sensitive to damage than cells with a lower passage. Cells react differently after NGF differentiation—cells with high passage are more sensitive to damage due to a toxic substance. Sakagami et al. also showed that cells as a result of differentiation become more resistant to drugs than undifferentiated cells (from 1.1 to over 10,000 times) [41]. Furthermore, a research team led by Kinarivala showed that the passage number also affects the phenotype of adherent cells [40].

The main disadvantages of PC12 cells, in addition to tumour origin, are non-development of synaptic endings even after 14-day differentiation and high morphological variability depending on the passage number. As the PC12 Adh line was derived from the traditional PC12 line as a result of multiple passages [3,20], the obvious conclusion is that with subsequent passages, cells grown in suspension become morphologically similar to PC12 Adh line. Based on this knowledge, we considered it appropriate to use cells with a passage number between 15 and 25 in our study.

In neurobiological studies on PC12 cells grown in suspension, poly-l-lysine (PLL), poly-d-lysine (PDL), fibronectin, laminin, or collagen (type I and IV) are usually used to coat the surface of culture vessels [33,34]. These compounds, except for PDL, occur naturally. Orłowska et al. investigated the effect of selected coatings (PLL, fibronectin, and laminin) and NGF concentration on differentiation processes of PC12 cells [33]. They have found that double coatings in the presence of the 100 ng/mL NGF not only led to the strongest cell attachment to the surface but also early stimulation of cell differentiation and neurite outgrowth was observed.

Cell adhesion after modification with polylysine occurs passively due to the attraction between the positively charged polymer and negatively charged cells. The disadvantage of PLL, which PDL does not have, is the lack of resistance to enzymatic degradation [37]. Collagens, which are triple-helical proteins composed of α-chains, form the structure of the extracellular matrix (ECM), providing a natural environment for cell growth and proliferation [42,43]. PC12 cells actively attach to collagen and laminin, which is also a component of ECM, in the presence of Mg^2+^ [44]. In our study for cells grown in suspension, we observed faster cytoplasm reorganisation and adhesion to the surface modified with type I collagen than PDL or PLL. Keshmirian noticed that PC12 cells after sowing on collagen behave as on a two-dimensional scaffold, thus they do not aggregate to each other, while on laminin coating the risk of cell aggregate formation increased with the seeding density [34]. Convertino presented a surface analysis with PLL, PDL and collagen layers, performed by atomic force microscope. Polylysine modified surface is more homogeneous, while network aggregates can be observed in the collagen coating [45]. Therefore, it can be assumed that the heterogeneity of the surface affects the stronger attachment of cells to the surface. Collagen occurs naturally in the ECM, hence it probably also has an impact on the differentiation of cell cultures. Surface modification with collagen, in contrast to PLL and PDL, allows the formation of ligand-receptor bonds, which allows faster reorganisation of the cytoskeleton and cell flattening (observed in our work as their polygonal shape). In our study, we also noted much longer neurites in the presence of NGF on collagen surfaces compared to polylysine ones. In addition, neurite outgrowth measured spectrofluorimetrically showed that for PC12 cells grown in suspension, the use of surface coating is more important than NGF administration. There are many studies indicating the high importance of cell adhesion to the substrate for the process of neurite growth. It is even thought that cell adhesion has a greater effect than the presence of NGF, which in turn is more important for the long-term stability of neurites [46]. In contrast, the surface coating or its lack is not very important for adherent PC12 cells.

After 24 h of cell adhesion and regeneration, differentiation was started using a medium containing NGF—a polypeptide neurotrophic factor that activates the tropomyosin receptor kinase A (TrkA) to facilitate cell transition from proliferation to differentiation. NGF affects biochemical, electrophysiological and morphological changes in PC12 cells, making them similar to sympathetic nerves. An important effector in NGF activity is ribosomal S6 kinase 1 (RSK1), which is sufficient to differentiate cells [47]. Under the influence of NGF, many biochemical changes occur: the number of dopamine receptors increases (which makes cells similar to midbrain dopaminergic neurons), sensitivity to acetylcholine increases, and potassium channels appear on cell membranes, which show electrical excitability [4,48,49]. During incubation of PC12 cells with NGF, the expression of the microtubule-associated protein 2 (MAP-2) important in neuritogenesis increases significantly (several times) [47]. Besides, there are receptors for advanced glycation end products (RAGE) in the cell membrane of differentiated cells [50]. All this confirms that only differentiated PC12 can constitute a model of nerve cells.

The incubation time in NGF-containing differentiation medium is selected by the researchers individually. Usually, it is from 2 to 14 days. In both PC12 lines tested, we observed an increase in neurite length and density between 2nd and 3rd day of incubation with NGF. In the suspension cells, the condition of neurites between 3rd and 5th day remained stable, and another marked increase of length and density occurred after the 5th day of incubation. In the case of the PC12 Adh line, we noticed that from the 7th day of incubation with NGF, the neurites were getting shorter, and the density decreased even from the 5th day. Furthermore, increased cell proliferation was observed on the 14th and 21st day. Drubin confirmed that during the first 2 days of incubation with NGF, there was no marked increase in microtubule mass, and after 3 days, neurite length increased even twice [51]. Das et al. noticed in their study that on the 6th day of incubation of PC12 cells in the presence of 50 ng/mL, the effect of NGF reached a plateau [11]. Unfortunately, they did not state in the paper which PC12 line was exactly used and the cell passage numbers.

Expression of the NeuN protein is observed for most adult neuronal cells, whereas the expression of DCX (doublecortin) is characteristic of immature developing cells of this type. The level of DCX expression for PC12 cells is significantly increased under the influence of NGF [47,52]. It is also known that this protein is an inhibitor of neurite growth [52]. In our results, we observed the expression of DCX and NeuN proteins for traditional PC12 cells, whereas, for adherent cells, only NeuN protein was expressed. We observed NeuN expression in both cell lines, but in traditional PC12 cells, expression was localised to cell nuclei, and in adherent cells, it occurred for the whole cell, both in the nucleus and the cytoplasm. For many years, NeuN was considered a specific marker that allows the recognition of adult neurons [38]. Recent studies, however, have shown that quantitative assessment of this protein does not give reliable information, because its expression occurs in many tissues as well as many cell lines that are not-neuronal cells [38,53,54]. Additional assessment is necessary, whether NeuN is expressed in or around the nucleus or in the cytoplasm. Cells expressing NeuN in both the nucleus and cytoplasm have also been reported, as in our study in the case of PC12 Adh cells. It was assumed that the NeuN protein, which is a product of the Rbfox3 gene, is a transcriptional regulator involved in neuronal differentiation [53]. However, it probably also has other still unknown functions, as indicated by its presence in cytoplasm of cells in many tissues.

In our work, we have shown that the concentration of NGF administered is of great importance for the degree of differentiation of both PC12 and PC12 Adh cells. Neurites were on average about two times longer after administration of 100 ng/mL NGF compared to 50 ng/mL. However, the concentration did not affect the average number of neurites in the cells (neurite density). Besides, for both cell lines, the effect of rat NGF at a concentration of 100 ng/mL was slightly stronger than the effect of human NGF. An innovative method of NGF administration was proposed by Bhang et al., who designed a collagen gel from which NGF was continuously released for up to 4 days of culture [55]. Comparison of the use of collagen gel loaded with at least 10 ng/mL NGF and traditional daily administration of 100 ng/mL NGF showed similar viability and differentiation of cells. They also checked cell differentiation at lower NGF doses and confirmed that the right NGF concentration is crucial for achieving proper differentiation.

## 5. Conclusions

Collagen coating was the best and most universal type of modification of the culture vessel surface for PC12 cells. This method was optimal for both cell lines. For PC12 Adh cells, the use of poly-d-lysine coating was equally effective.

Both the PC12 (ATCC CRL-1721) and PC12 Adh (ATCC CRL-1721.1) cells underwent differentiation after NGF treatment, with the level of development of neuronal features highly dependent on NGF concentration. Besides, the effect of rat NGF was slightly stronger than the effect of human NGF.

For suspension cells, the best neuronal characteristics (length and density of neurites) is observed after 14 days of incubation with 100 ng/mL NGF (change every 48 h), while for adherent cells after 3–5 days, after which they began to proliferate.

In the PC12 cell line, doublecortin (DCX) expression in the cytoplasm and NeuN in the cell nucleus were found. In turn, in the PC12 Adh line, DCX was not expressed, and NeuN expression was located in the entire cell (both in the nucleus and cytoplasm). Combined with recent articles questioning the usefulness of the NeuN protein as a neuronal marker, this suggests avoiding considering PC12 Adh cells as a nerve cell model.

## Figures and Tables

**Table 1:** Differences between PC12 and PC12 Adh cell lines [20,21,24,25].

Feature	PC12 Cell Line (ATCC CRL-1721)	PC12 Adh Cell Line (ATCC CRL-1721.1)
Cell type	Cluster of floating cells	Adherent cells
Morphology	Small and irregular shape	Polygonal shape
Culture medium	RPMI-1640 with 10% DHS, 5% FBS	Ham’s F-12K with 15% DHS, 2.5% FBS
Differentiation/neurite outgrowth	Differentiation with NGF or Opti-MEM with 0.5% FBS	Neurite outgrowth promoted by Rho kinase (ROCK) inhibition
